# Expression, Regulation, and Function of β-Defensins in the Bovine Mammary Glands: Current Knowledge and Future Perspectives

**DOI:** 10.3390/ani13213372

**Published:** 2023-10-31

**Authors:** Mojtaba Daneshi, Joel S. Caton, Luciano S. Caixeta, Zohre Eftekhari, Alison K. Ward

**Affiliations:** 1 Department of Animal Sciences, Center for Nutrition and Pregnancy, North Dakota State University, Fargo, ND 58108, USA; 2 Department of Veterinary Population Medicine, University of Minnesota, St. Paul, MN 55108, USA; lcaixeta@umn.edu; 3 Biotechnology Department, Pasteur Institute of Iran, Tehran 1316943551, Iran; z_eftekhari@pasteur.ac.ir; 4 Department of Veterinary Biomedical Sciences, University of Saskatchewan, Saskatoon, SK S7N 5B4, Canada; alison.ward@usask.ca

**Keywords:** antimicrobial peptides, mastitis, cattle, NF-κB, innate immune, udder, transcription factors, antimicrobial resistance, vitamin D

## Abstract

**Simple Summary:**

Mastitis, a common ailment affecting dairy cows, carries significant economic implications. Common treatments with synthetic antimicrobials face challenges due to the emergence of antimicrobial resistance. This article focuses on a vital component of the immune system: bovine β-defensin peptides. These naturally-occurring antimicrobial agents produced by cows exhibit strong activity against pathogens that cause mastitis. The efficiency of these peptides in preventing and treating infections of the mammary glands can be improved by knowing how they are controlled and what part they play in the immune system. This study tries to illuminate a possible route for creating novel, natural, antibacterial treatments for dairy cattle in the future and minimizing dependency on synthetic antimicrobials. Furthermore, by exploring the expression routes and factors affecting β-defensin expression, the potential to improve immunity and reduce the need of synthetic antimicrobials is emphasized. We also point out areas that need more research and indicate current gaps in the literature.

**Abstract:**

β-Defensins are cationic antimicrobial peptides (AMPs) that play an important role in the innate immune defense of bovines. They are constitutively expressed in mammary glands and induced differently in response to pathogens. Their expression is influenced by various factors, including hormones, plant-derived compounds, and dietary energy imbalance. The toll-like receptors (TLRs)/nuclear factor-kappa B (NF-κB) pathway plays a crucial role in β-defensin induction, while alternative pathways such as mitogen-activated protein kinase (MAPK) and epigenetic regulation also make substantial contributions. β-Defensins exhibit bactericidal activity against a wide range of pathogens, including two major mastitis pathogens, *Escherichia coli* (*E. coli*) and *Staphylococcus aureus* (*S. aureus*), primarily through membrane disruption. β-Defensins have low cytotoxicity to host cells and demonstrate immunomodulatory properties, and pathogens also display minimal resistance to these AMPs. Given the increasing concern in antimicrobial resistance, the potential of β-defensins as natural antimicrobials has garnered considerable attention. This article provides an overview of the characteristics of bovine β-defensins, their expression pathways, their mode of action, and factors influencing their expression in the mammary glands of cattle. Additionally, it identifies the current gaps in research within this field and suggests areas that require further investigation. Understanding the regulation and function of β-defensins offers valuable insights to develop effective strategies for strengthening the immune system of mammary glands, reducing the reliance on synthetic antimicrobials, and explore novel natural antimicrobial alternatives.

## 1. Introduction

Mastitis, a common infectious condition that affects the mammary glands of dairy cows, results in significant financial losses due to a drop in milk production and altered milk composition, as well as treatment expenses [1,2,3]. The pooled prevalence of subclinical mastitis and clinical mastitis were reported to be 42% and 15% world-wide, respectively [4]. Currently, synthetic antimicrobial agents, administered through intramammary infusion or parenteral administration, are the most common treatments for mammary gland infections in dairy cattle. However, the advent of microorganisms that are resistant to these antimicrobials has sparked considerable debate over their usage [5,6,7]. Antimicrobial resistance in animals can jeopardize the effectiveness of treatments and the animal industry’s sustainability. Furthermore, the emergence of drug-resistant infections arising from the use of antimicrobials in veterinary medicine poses a substantial and grave threat to human health [8,9]. Hence, there is a need in the dairy industry to discover effective alternative antimicrobials with broad-spectrum properties [9,10]. Furthermore, leveraging the biological capabilities of cattle to boost their immune system against infections of the mammary glands is another path that should be explored. This approach requires a comprehensive understanding of the immune system and how it regulates the inflammatory response and the clearance of pathogens in the mammary gland.

Antimicrobial peptides (AMPs) are a crucial component of the bovine innate immune system, which serves as the first line of defense against pathogens in the mammary glands [11,12]. Among these AMPs, bovine β-defensin peptides are a crucial subset, exhibiting high bactericidal activity against bacteria responsible for mastitis in dairy cattle, such as *Escherichia coli* (*E. coli*) and *Staphylococcus aureus* (*S. aureus*) [13,14,15]. Since AMPs act as the first line of defense against various pathogens, understanding the mechanisms that control their expression and activity in the mammary glands will enable us to maximize their efficiency, efficacy, and enhancement of their expression when cattle are most vulnerable to microbial infections. Additionally, they may be used to introduce novel natural and synthetic antimicrobial agents that could help prevent and treat clinical and subclinical mammary glands infections. It is important to note, however, that the use of AMPs as auxiliary therapeutic agents or drugs will require some modifications and strengthening in order to possess appropriate therapeutic properties, such as metabolic stability, high affinity and specificity for a particular enzyme or receptor. To accomplish these goals, therefore, it is crucial to have integrated and comprehensive information on AMPs, aiding us to take the next steps towards developing effective AMP-based therapeutics.

To date, many articles have been published on the structure and function of AMPs in various tissues. However, to our knowledge, β-defensins in the mammary gland of cattle have not been thoroughly scrutinized. The objectives of this article are categorized into four approaches: (1) Providing background information on these AMPs, (2) describing the potential mechanisms underlying their expression and activity, (3) discussing factors that influence their regulation, and (4) proposing potential areas for future research. By shedding light on these aspects, we aim to deepen our understanding of the role of β-defensins in the mammary glands and pave the way for the development of novel approaches for combating mastitis in dairy cattle, a decreased reliance on synthetic antimicrobials, and promotion of the development of new natural antimicrobial components utilizing AMPs.

## 2. Literature Search and Selection

We conducted a narrative review of the literature on β-defensins in the bovine mammary gland. To be included, articles had to meet the following criteria: they had to be written in English, accessible in full-text format, complete, and directly relevant to our topic. We searched PubMed and Scopus in October 2021 using the following keywords: (“Antimicrobial peptides” OR “β-defensins”) AND (“cattle” OR “cow”) AND (“udder” OR “mammary gland” OR “teat”). A total of 223 articles were retrieved based on their titles, abstracts, and publication dates (we only included publications after 1992 because β-defensins in bovine were first discovered in 1993 [14]).

We pooled the results from the database and used MENDELEY^®^ software (Mendeley Desktop, version 1.19.8, Elsevier^©^) to filter out duplicate articles, resulting in 169 unique articles. We then read the full texts of these articles in detail and selected 81 articles that were relevant to our topic, excluding those that did not relate to β-defensins or cattle. In June 2023, an additional search was performed using Google Scholar, PubMed, and Scopus to identify recently published papers. From this, we identified and included nine additional articles directly related to our topic in our review. During the writing process, we also incorporated 18 more references to provide clearer context and support for our arguments.

## 3. General Aspects of β-Defensins

### 3.1. Structure and Classification of β-Defensins

AMPs act via innate immunity and are classified based on charge, sequence, and structure. Defensins are a type of cationic AMP and have a three-dimensional β-sheet structure [16,17]. They belong to a family of small peptides and are characterized by their high cysteine and arginine content. The structure of defensins is stabilized by three disulfide bonds, which are formed by six highly conserved cysteine residues [11,18]. Based on the location of the disulfide bonds between cysteine residues, defensins are classified into three subfamilies: α- (C1–C6, C2–C4, and C3–C5 residues), β- (C1–C5, C2–C4, and C3–C6 residues), and θ-defensins (C1–C6, C2–C5, and C3–C4 residues) [19]. Among these subfamilies, α-defensins are found in a wide range of mammalian species, while θ-defensins are exclusively found in nonhuman primates. Interestingly, bovine genomes do not contain α-defensins [20,21]. The disulfide bonds within β-defensin molecules are crucial for their proper folding and stability, which ultimately enable their antimicrobial activity. These bonds also contribute to the structural integrity of the peptide and can have additional roles in cell signaling and immunomodulation mediated by β-defensins [22]. It is also hypothesized that the primary role of the disulfides is to shield the backbone of β-defensins against proteolysis in environments containing protease [23].

### 3.2. Genomic Organization and Expression of Bovine β-Defensins

The β-defensin gene family is the largest in bovine compared to other mammals, providing insights into genomic organization and expression. These β-defensin genes are distributed among four distinct gene clusters located on chromosomes 8 (cluster A), 13 (cluster B), 23 (cluster C), and 27 (cluster D), coding for 4, 19, 5, and 30 genes, respectively. Within each gene cluster, the sequences of β-defensin genes exhibit greater homology to each other compared to genes in other clusters. Genes on chromosomes 8 and 23 have not yet been studied for function or expression. Expression of the bovine β-defensin genes located on chromosome 13 are restricted to testis, though two particular β-defensins, β-defensin 123 and 124 (BBD123 and BBD124), have been detected at lower levels in mammary cells extracted from milk. Cluster D encompasses 16 well-characterized β-defensins, which range in length from 38 to 42 amino acids. Notable members of this cluster include bovine neutrophil β-defensins 1-13 (BNBD1-13, also known as DEFB1–13), enteric β-defensin (EBD), lingual antimicrobial peptide (LAP), and tracheal antimicrobial peptide (TAP) [19,22,24,25]. Among these, BNBD5 [26,27] and LAP [28,29] have been found to be the most abundantly expressed in the mammary glands.

### 3.3. Mechanisms of Action: Interactions and Activities of β-Defensins

Cationic and amphipathic structures allow β-defensins to effectively interact with biological membranes. The antimicrobial mechanisms of these compounds are via membrane disruption and permeabilization of the target [21,30], leading to the direct bactericidal activity against a variety of pathogens including major the mastitis pathogens *E. coli* and *S. aureus* [31,32]. However, it has been reported that *Prototheca wickerhamii*, an agent of granulomatous mastitis in cattle, when treated with LAP did not exhibit apparent surface damage in scanning electron microscopy analysis, indicating the existence a novel non-lytic mechanism of β-defensins [15]. It has been demonstrated in many studies that AMPs show a preference for interacting with target microbes rather than with host cells. The basis for this is the fact that the cytoplasmic membranes of prokaryotic and eukaryotic cells differ significantly in terms of composition and topological arrangement of lipids. Additionally, prokaryotic cells typically exhibit a transmembrane potential of −140 mV, which contrasts with the less negative membrane potential of eukaryotic cells (−15 mV). Consequently, due to electrostatic interactions between positive charges on cationic peptides and negative charges on lipids, cationic peptides prefer to bind to bacteria [33]. Thus far, only the cytotoxicity of LAP on bovine mammary epithelial cells (MECs) has been studied, revealing no significant cytotoxic effects [15]. However, the cytotoxicity of other β-defensins towards bovine MECs remains unexplored. These peptides have shown efficacy against *E. coli* by employing both oxygen-dependent and oxygen-independent mechanisms. Given the low oxygen partial pressure observed in infected tissues, the contribution of oxygen-independent mechanisms to bacteriolysis becomes particularly significant [14,34,35]. It is worth mentioning that a β-defensin isolated from buffalo polymorphonuclear cells possessed antiviral activities against Rinderpest Virus and Newcastle Disease Virus [36]. However, to the best of our knowledge, no investigation has been conducted into the role of bovine β-defensins in resisting viral infections in cattle.

In addition to their roles in host defense, β-defensins also exhibit immunomodulatory properties, including the ability to stimulate chemotaxis [22,37]. For instance, research has shown that β-defensins attract immature bovine dendritic cells, with BNBD3, BNBD9, and EBD demonstrating the highest chemotactic activity [38]. Furthermore, these peptides have been found to induce both pro-inflammatory and anti-inflammatory cytokine responses, such as the secretion of tumor necrosis factor α (TNF-α), interleukin (IL)-1β, and IL-10 in macrophages [39]. Despite the robust evidence supporting the immunomodulatory effects of β-defensins, no studies have investigated their specific role in immunomodulation within the bovine mammary gland, which is a critical site susceptible to infections and inflammation. Hence, there exists a pressing requirement to investigate and clarify the immunomodulatory impacts of β-defensins in the mammary gland of cattle, both through in vivo and ex vivo approaches.

## 4. Expression of β-Defensins in the Mammary Glands

### 4.1. The Spatial Expression Pattern of β-Defensins

**Figure 1:**
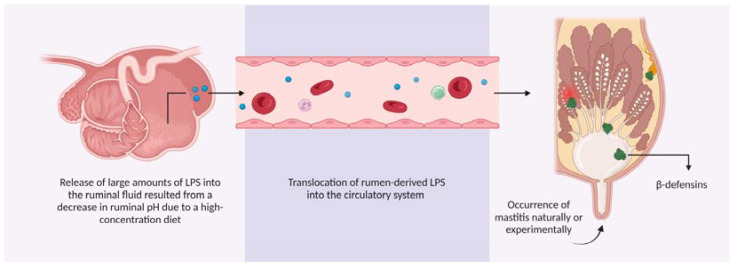
β-Defensins expression in the parenchyma, MECs, and mammary lymph nodes of healthy bovine mammary glands and in response to mastitis-causing pathogens or their virulence factors, both in natural and experimental conditions. Additionally, the release of LPS from rumen to the bloodstream due to a high-concentrate diet leads to the expression of β-defensins in the mammary glands.

β-defensins are constitutively expressed in the parenchyma, MECs, and lymph nodes of healthy bovine mammary glands, regardless of parity, age [26,29,40], or lactational status [19]. They are also expressed in milk [13,18,41,42]. Having an exposed position to pathogens and an abundance in healthy mammary glands, MECs play an important role in udder immunity. The epithelial cells are where β-defensins mRNA [26,28,29] and protein [43,44] are expressed predominantly, implying that these cells secrete this protein into milk. There is also no difference in the expression of these β-defensins among quarters in each individual cow [45]. In addition to being synthesized predominantly in MECs, β-defensins are highly expressed in monocytes, macrophages, and neutrophils, which migrate into the mammary gland during mastitis [46,47]. Production of β-defensins is also induced in response to infection [26,40,48,49,50], as well as lipopolysaccharides (LPS) and lipoteichoic acid (LTA) challenge [51,52,53], or in response to the translocation of rumen-derived LPS into the circulatory system due to high-concentrate diets [54,55] (Figure 1). It was assumed that the induction of β-defensin genes is not systemic [26], but rather spatially regulated and confined to the infected quarter [56,57]. However, evidence challenges this view, indicating that β-defensins are induced in neighboring quarters of the infected quarters [48,58]. Moreover, recent investigations have found increased expression of β-defensins in circulating leukocytes [59,60] and serum [49] of cattle with clinical mastitis but not in those with subclinical mastitis (Supplementary Table S1). It is not known how the transcriptome of uninfected quarters and circulating leukocytes are affected by the infection of quarters.

### 4.2. Pathogen-Specific Expression Patterns of β-Defensin

The expression levels of β-defensin genes differ according to the pathogens that cause mastitis. Studies have shown that *E. coli* and LPS invariably cause the β-defensins in the udder and circulating leukocytes to be upregulated [60] (Supplementary Table S1). Conceivably, the magnitude and kinetics of LAP mRNA expression increases almost independently from *E. coli* dose in bovine MECs [61]. Compared to *S. aureus* and LTA, the increase in expression of β-defensin in response to *E. coli* [56,62,63] and LPS [52,64] is more significant and sustained. However, the administration of an enterotoxigenic *E. coli* vaccine suppresses the secretion of LAP in milk [65]. On the contrary, *S. aureus* and LTA not only fail to enhance these peptides [56,62,66,67], but they can also lead to the downregulation of β-defensins [11,68] (Supplementary Table S1). This may account for one possible reason for chronic and localized *S. aureus* infection in the udder. Furthermore, it has been reported that the levels of expression of DEFB1, BNBD4, and BNBD5 genes are higher in tissues obtained from infected quarters with coagulase-positive *Staphylococci* compared to those with coagulase-negative *Staphylococci* [40]. *Mycobacterium bovis* (*M. bovis*), which is one of the most serious contagious pathogens affecting cattle, has a minor impact on the expression of β-defensins [63]. Out of all bovine viruses, only the Bovine Leukemia Virus has been studied in terms of its relationship with β-defensins in the udder. This research suggests that there is a negative association between the concentration of LAP in milk and the proviral load of the Bovine Leukemia Virus, which indirectly contributes to the development of bovine mastitis [69].

### 4.3. Effect of Plant-Derived Compounds on the Expression of β-Defensins

The expression of β-defensins can be increased by γ-thionin, a plant β-defensin that is sourced from *Capsicum chinense* [10]. Another study investigating the effects of *Carica papaya* supplementation in cattle demonstrated the capability to upregulate expression of BNBD13 in milk somatic cells [70]. Conversely, novel compounds derived from plants, such as the 2,3-dihydro-flavonoid drug farrerol extracted from *rhododendron*, have been shown to downregulate the mRNA expression of specific β-defensins (TAP and BNBD5) in bovine MECs [71]. These initial findings, taken together, provide promising insights into the potential use of plant-based interventions to enhance antimicrobial defenses.

## 5. Mechanisms of Expression

### 5.1. NF-kB Pathway

The nuclear factor-kappa B (NF-κB) pathway plays a crucial role in the regulation of β-defensin expression in the mammary gland of cattle. Pathogens are identified by pattern recognition receptors (PRR) such as the well-characterized family of toll-like receptors (TLR) [26,72]. For example, TLR2 is activated by LTA of Gram-positive bacteria, while TLR4 is triggered by LPS of Gram-negative bacteria [57,73,74], even though in the udder they are regulated similarly and in concert during the process of mounting an immune response [26,74]. Upon activation of TLR, transcription factor families such as NF-κB and activator protein-1 (AP-1) are ultimately activated [72,75]. The NF-κB family of transcription factors are involved mainly in immune and inflammatory responses [76]. They are comprised of five different factors: NF-κB1 (also known as p50 or p105), NF-κB2 (also known as p52 or p100), p65 (also known as RelA), RelB, and c-Rel. An inactive form of these substances is stored in the cytoplasm, combined with regulatory proteins called inhibitors of κB (IκB). Upon phosphorylation of IκB by IκB kinase (IKK), NF-κB is released and translocated into the nucleus, leading to gene activation [77,78]. A substantial level of active NF-κB factors, which are predominantly NF-κB p50 homodimers [72], is already available in sterile udders of healthy cows, leading to the constitutive expression of β-defensins [52,57], while stimulation preferentially recruits NF-κB-p65 to the LAP promoter, leading to a transition in the balance state from p50 homodimers predominantly occupying the resting promoter to heterodimers (NF-κB-p65, -p50) on the fully activated promoter [72]. Notably, there is a close correlation between the transcription of β-defensin genes and the intracellular accumulation of the translated peptides [25]. During infection, chromatin decompaction occurs at the LAP promoter, allowing for NF-κB p65 to bind and stimulate LAP expression [72]. To support this, it has been observed that *E. coli* [26,56,74,79] and LPS [73,80] increase TLR mRNA and β-defensin expression. The presence of *E. coli* and LPS in bovine MECs also causes higher expression of phospho-p65 and phospho-IκB [74,81,82,83], as well as the downregulation of IκBα [72]. Peroxisome proliferator-activated receptor (PPARγ) inhibits NF-κB transfer into the nucleus by a physically interaction and is downregulated substantially by *E. coli* stimulation [81]. Furthermore, the presence of LPS systematically in the bloodstream is accompanied by increased phosphorylated NF-Κb and phosphorylated IκBα, resulting in upregulation of LAP protein expression in mammary glands [54].

Conversely, disruption of the TLR/NF-κB pathway or inhibition of NF-κB activation can prevent the expression of β-defensins, such that mutations in the core of the NF-κB binding sites greatly lowered basal expression of LAP and abolished inducibility [72]. For instance, acetylsalicylic acid, which is a specific inhibitor of IKK-β activity, prevents the activation of NF-κB genes, leading to drastic downregulation expression of TAP in bovine MECs [11,68]. Another factor that influences the activity of the LAP promoter is the CAAT box enhancer binding protein β (C/EBPβ). C/EBPβ has an antagonistic physiological role against NF-κB p65 and has been shown to decrease the activity of the LAP promoter [72].

**Figure 2:**
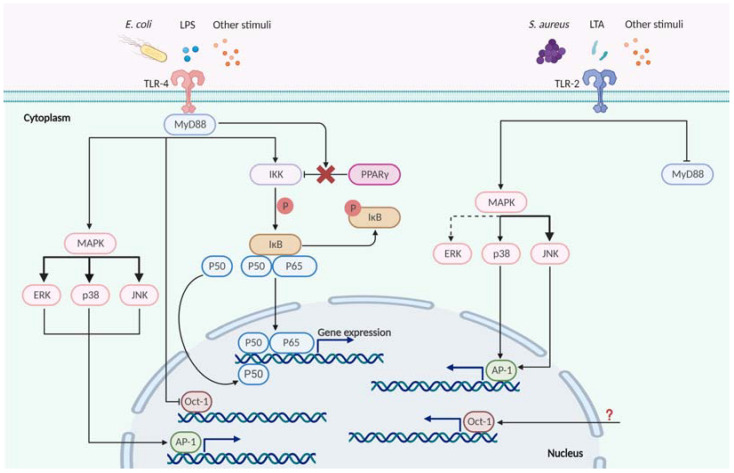
NF-κB, MAPK and Oct-1 pathways for expression of β-defensins. NF-κB can be triggered by various stimuli, such as *E. coli* and LPS. Upon activation, IKK phosphorylates IκB kinase, resulting in the phosphorylation and subsequent degradation of IκB. Additionally, PPARγ inhibits the translocation of NF-κB into the nucleus through physical interaction, and *E. coli* significantly diminishes this interaction (cross sign). This process releases the NF-κB dimer, allowing it to move into the nucleus. In the udders of healthy cows, there exist a notable amount of active NF-κB factors, primarily NF-κB p50 homodimers, contributing to the continuous expression of β-defensins. Stimulation predominantly recruits NF-κB-p65, altering the balance from primarily p50 homodimers occupying the resting promoter to heterodimers (NF-κB-p65, -p50). *S. aureus* deactivates the TLR-4/MyD88/NF-κB axis (represented by a T-line) by obstructing MyD88-dependent signaling, which serves as the principal regulator of the TLR signaling pathway. Exposure of MECs to LPS triggers a robust activation of the MAPK pathway (depicted by thick arrows). This activation includes the phosphorylation of MAPK components, JNK1/2, ERK1/2, and p38, which correlates with an increase in β-defensin expression. *S. aureus* activates JNK1/2 (thick arrow), reduces the phosphorylation of ERK1/2 (dashed arrow), while exhibiting no impact on p38 MAPK levels (solid arrow). Stimulation of the MAPK pathway can lead to the activation of AP-1. Oct-1 operates as a transcription factor in the regulation of β-defensin expression. However, it remains unresponsive to LPS stimulation (indicated by the T-line), and the cascade associated with this process has not yet been explored (indicated by a red question mark). AP-1 = Activator Protein-1; *E. coli* = *Escherichia coli*; ERK = Extracellular Signal-Regulated Kinases; IκB = Inhibitors of κB; IKK = IκB Kinase; JNK = Jun Amino-Terminal Kinases; LPS = Lipopolysaccharides; LTA = Lipoteichoic Acid; MECs = Mammary Epithelial Cells; MAPK = Mitogen-Activated Protein Kinase; MyD88 = Myeloid Differentiation Primary Response 88; NF-κB = Nuclear Factor-Kappa B; Oct-1 = Octamer Transcription Factor-1; P = Phosphorus; PPARγ = Peroxisome Proliferator-Activated Receptor Gamma; *S. aureus* = *Staphylococcus aureus*; TLR = Toll-Like Receptor.

As mentioned earlier, induction of β-defensin expression by *S. aureus* is variable (Supplementary Table S1). This raises a question of whether *S. aureus* would exploit the TLR/NF-kB pathway. Studies have shown that *S. aureus* can induce increased NF-κB p65 and decreased cytoplasmic NF-κB p65 levels [84], as well as decreased PPARγ expression [75]. However, many studies have established that *S. aureus* fails to activate NF-κB [68,74,85,86,87]. This lack of activation stems from the inhibition or slight regulation of TLR by the pathogen [48,56,79]. Specifically, *S. aureus* inactivates the TLR4/myeloid differentiation primary response 88 (MyD88)/NF-κB axis via blocking MyD88-dependent signaling, which is the primary regulator of the TLR signaling pathway (Figure 2). Collectively, it becomes difficult to assume that *S. aureus* induces β-defensins via the NF-κB pathway. It is conceivable that *S. aureus* circumvents the innate immune response and localizes in the udder through the mechanisms described above. In addition to *S. aureus*, live *M. bovis* has been shown to prevent the expression of β-defensins by inhibiting TLR2 and TLR4 mRNA expressions in bovine MECs. This inhibition may explain the immune evasion strategy employed by *M. bovis* [63].

### 5.2. MAPK Pathway

The mitogen-activated protein kinase (MAPK) signaling pathway has been implicated in the induction of β-defensins. This pathway consists of three subfamilies: jun amino-terminal kinases (JNK), extracellular signal-regulated kinases (ERK), and p38, all of which can be activated by TLR stimulation [75,83]. In the case of LPS, exposure of bovine MECs triggers a robust activation of the MAPK pathway (Figure 2). This includes the phosphorylation of MAPK components, JNK1/2, ERK1/2, and p38 which is associated with a surge in the expression of β-defensins [83]. This may explain the higher induction of β-defensins observed in response to *E. coli* compared to *S. aureus*. Conversely, in the context of *S. aureus* infection in bovine MECs, the involvement of the MAPK pathway appears to be a subject of some ambiguity. The activation of JNK1/2 and the reduction in phosphorylation of ERK1/2 is observed, while p38 MAPK levels remain comparatively stable (Figure 2). Although this intricate MAPK response coincides with the increased expression of specific β-defensins in response to *S. aureus*, the majority of other β-defensins remain unaffected, highlighting the selective nature of this pathway and its potential role in the regulation of β-defensins [66,75]. It should be noted that stimulation of the MAPK pathway is known to lead to the activation of various transcription factors, including AP-1. Given that specific β-defensins (LAP, TAP, BNBD5, and BNBD10) contain potential transcription binding sites for AP-1, this provides a plausible mechanism for the selective induction of these β-defensins through the MAPK/AP-1 axis during encounters with *S. aureus* [75]. However, the potential utilization of the MAPK pathway by other mastitis-causing pathogens, such as *E. coli*, to induce β-defensins expression within the mammary glands remains uninvestigated.

### 5.3. DNA Methylation and Histone Deacetylation

The expression of β-defensins in bovine MECs is significantly influenced by epigenetic mechanisms, particularly DNA methylation and histone deacetylation [88]. Conversely, inhibition of DNA methyltransferase and histone deacetylase activities can induce an open chromatin state, resulting in increased expression of β-defensins in bovine MECs [83,88,89]. Histone deacetylase was discovered to be downregulated by *E. coli*, resulting in an elevated expression of β-defensins, whereas this effect was not observed with *S. aureus* [61]. An intriguing finding is the synergistic effect observed when inhibiting DNA methyltransferase alongside LPS treatment. Combining LPS stimulation with 5-aza-2′-deoxycytidine (an inhibitor of DNA methyltransferase) further enhances the expression of β-defensins compared to either treatment alone [88]. In contrast, studies have revealed that *M. bovis* causes a decrease in histone demethylase activity. This reduction in histone demethylase activity may contribute to the inability of *M. bovis* to induce the expression of β-defensins [63].

### 5.4. Octamer Transcription Factor-1

Another transcription factor involved in the regulation of LAP and TAP expression is octamer transcription factor 1 (Oct-1) [90]. This transcription factor plays a role in both general transcriptional processes by opening chromatin and in transducing extracellular signals through its interactions with different partners [91]. A study using promoter serial deletion experiments has demonstrated the positive regulation of LAP and TAP gene expression by Oct-1, both for initiating and maintaining transcription. However, the involvement of Oct-1 in the immune response against Gram-negative bacteria seems to be limited, as stimulation with LPS did not result in an increase in Oct-1 protein levels [90] (Figure 2). However, it is crucial to conduct further research into the Oct-1 pathway cascade, also explore the potential association between the Oct-1 pathway and Gram-positive bacteria.

## 6. Factors Influencing the Expression of β-Defensins in Bovine Mammary Glands

### 6.1. Vitamin D

Vitamin D has a direct immunomodulatory effect in innate immune cells [80,92] and the mammary gland [46] of cattle. There is, however, a paucity of evidence supporting vitamin D-mediated expression of β-defensins in the bovine mammary gland [46,47,80,88,93]. In fact, vitamin D signaling may even suppress the response of certain β-defensins in bovine MECs to LPS stimulation [27,80]. Only BNBD4 and BNBD7 have been identified as β-defensins upregulated in response to vitamin D [27,46,80,88]. Although the exact mechanisms by which vitamin D regulates β-defensin expression in the mammary gland of cattle are not well understood [47,80], it appears that the upregulation of certain β-defensins is indirect and requires an intermediate factor induced by vitamin D [80]. On the other hand, the inhibitory effect of vitamin D on NF-κB activation may explain the downregulation of some β-defensins [27]. It is worth noting that, apart from vitamin D, there is a dearth of investigations on the effects of other micronutrients on the regulation of β-defensin genes expression in the bovine mammary glands.

### 6.2. Butyrate

A distinctive feature of bovine milk fat is the existence of butyrate [89]. Sodium butyrate can induce β-defensins mRNA expression in bovine MECs [75,83,89] via multiple pathways. It stimulates bovine MECs through the TLR2/p38 pathway, which leads to the activation of transcriptional factors such as AP-1 [75]. Furthermore, sodium butyrate has been shown to possess histone deacetylase inhibitor activity [94,95]. Its presence alone [82] and with *S. aureus* [89] induces histone acetylation, resulting in enhanced expression of β-defensins. Sodium butyrate with LPS, induces β-defensin expression in MECs via histone acetylation, as well as p38, JNK, and ERK1/2 MAPK pathways in bovine MECs. As a result, by boosting the innate immune response, sodium butyrate plays an effective role in the clearance of pathogens [83]. However, it has been observed that sodium butyrate can diminish the activity of certain transcriptional factors, including ERK1/2, JNK1/2 [75], and NF-κB [82,83]. The inhibitory mechanisms of sodium butyrate on these factors in bovine MECs are not yet fully understood. In nonruminants, butyrate has been found to inhibit phosphorylation and degradation of IκB, thereby preventing the release of NF-κB [96].

### 6.3. Other Short-Chain Fatty Acids

Sodium propionate and sodium hexanoate have been shown to upregulate TAP mRNA expression, while not affecting BNBD5 [97]. On the contrary, sodium acetate causes TAP and BNBD5 downregulation and upregulation, respectively [84]. Sodium octanoate, a medium chain fatty acid, differentially modulates β-defensin genes expression. It increases BNBD4, LAP, and BNBD10 mRNA expression, while decreases BNBD5 and does not affect TAP [98]. Nevertheless, the mechanism by which these fatty acids modulate the expression of β-defensins in bovine MECs are yet to be fully elucidated.

### 6.4. Conjugated Linoleic Acid

Conjugated linoleic acid (CLA), primarily in the cis-9, trans-11 form, is naturally found in ruminant milk and meat and has been shown to enhance the immune system [99]. However, it has been found that c-9, t-11 CLA downregulates BNBD5 expression in bovine MECs treated with *E. coli*. Remarkably, suppression of TLR4, phospho-IκB and NF-κB p65 related to the NF-κB signaling pathway and activation of PPARγ by c-9, t-11 CLA can be considered the explanations of the mechanisms by which c-9, t-11 CLA lower BNBD5 expression [81].

### 6.5. Prolactin

The effect of prolactin on β-defensin gene expression remains controversial. On one hand, prolactin alone has been shown to activate NF-κB and promote the expression of the TAP gene [68]. On the other hand, prolactin can significantly downregulate β-defensins [11,100]. Moreover, when prolactin is combined with *S. aureus* to mimic an infection in vivo, there is a drastic reduction in NF-κB and β-defensin genes expression in bovine MECs. This suggests that *S. aureus* can override prolactin-induced NF-κB activation, leading to intracellular persistence of the pathogen and the occurrence of chronic subclinical infections due to a weakened immune response [11,68,100]. Despite the induction of NF-κB by prolactin, the precise mechanism by which it downregulates β-defensins remains unknown.

### 6.6. Estradiol

Studies have shown that 17β-estradiol (E2) alone can upregulate the expression of a few β-defensins, such as DEFB1 and BNBD5, ex vivo experiments. However, E2 alone or in the presence of *S. aureus* is unable to enhance the expression of LAP, TAP, BNBD4, and BNBD10 [66]. Additionally, the concentration of LAP in milk does not significantly differ during the estrus cycle and ovulation synchronization protocol, indicating that E2 may not play a substantial role in its regulation [101]. The lack of TLR2 activation by E2 prevents the activation of downstream transcription pathways, including p38 phosphorylation and ERK, which are necessary for the induction of the majority of β-defensins [66].

The endocrine system has long been recognized for its central role in various aspects of mammary glands development, lactation onset, and maintenance [102]. Beyond prolactin and E2, hormone effects on β-defensin expression in cattle mammary glands are understudied. Further research is needed to explore the influence of hormones like progesterone, growth hormone, and calcitonin on β-defensin expression.

## 7. β-Defensins as Biomarkers

Several studies have investigated the potential of β-defensins as markers for mastitis. One such marker is LAP, which has been found to exhibit a significant positive correlation with somatic cell count (SCC) as determined by ELISA in milk [18,45,103]. This relationship has also been observed using in situ hybridization, revealing a substantial positive association between SCC and LAP expression in the mammary gland [28]. As previously discussed, the production of LAP is stimulated when MECs interact with pathogens through TLR, leading to the activation of NF-κB and subsequent production of IL-8 and LAP [18,72]. The release of IL-8 attracts neutrophils to the alveoli, which account for approximately 90% of total somatic cells during mastitis [47]. Neutrophils have been identified as a significant source of β-defensins during mastitis, particularly in response to LPS [47,80]. Their increase also coincides with the time of leukocyte infiltration [104]. Therefore, the concentration of LAP in milk increases as SCC increases [18,72]. However, LAP concentration tends to return to baseline levels sooner than SCC post-inflammation. This suggests that LAP accumulation within epithelial cells and its production because of stimulation may have a role in the initial phases of the immune response [51,64]. Consequently, measuring LAP may primarily aid in detecting the early phases of inflammation [18,72], rather than the late stages of recovery. In addition to LAP, recent research has shed light on the potential of another β-defensin, DEFB4, as a marker for mastitis [49]. This study demonstrated a significant difference in DEFB4 levels between acute clinical mastitis and subclinical mastitis, both locally and systemically, indicating its potential use as a marker for the detection of subclinical mastitis.

Although SCC is a commonly used method for monitoring udder health, it exhibits low sensitivity for diagnosing mastitis [105]. Therefore, combining β-defensins and SCC findings may offer a more rapid and accurate diagnosis of mastitis stage, providing a comprehensive assessment of the udder health and resulting in time and cost savings [18]. However, it is important to note that β-defensins are consistently elevated in response to *E. coli*, whereas their response to *S. aureus* is variable (Supplementary Table S1). Therefore, conducting further studies, large-scale β-defensin measurements, and standardization is crucial for establishing precise thresholds.

β-defensins have also been shown to have potential as a marker for predicting intramammary infection status at calving and for implementing blanket dry-cow therapy or selective therapy during the dry period. A prospective observational study found that mammary glands with no new infections at calving had significantly higher expression of β-defensin mRNA at 14 days pre-calving compared to those that developed new intramammary infections [106]. All together, they suggest that low or non-responsiveness of this part of the innate immune system can increase the risk of intramammary infection [106].

## 8. Knowledge Gaps for Clinical Application of β-Defensins

In recent years, AMPs have gained significant attention for their therapeutic potential. This is evidenced by the Food and Drug Administration (FDA) approving several AMPs as pharmaceuticals, including Bacitracin, Dalbavancin, Vancomycin, and Enfuvirtide [107]. While the primary focus of this paper was not on pharmaceutical applications, we aimed to highlight the potential of these defensins as natural based or synthetic forms antimicrobial agents that could be instrumental in preventing and treating clinical and subclinical mammary gland infections. Therefore, we explored β-defensins in bovine mammary glands, particularly their mechanisms of expression. However, when discussing the potential commercialization and clinical application of β-defensins for use in bovine mammary glands, based on the content of this review article and to the best of our knowledge, several challenges emerge:

Modification and Strengthening: As discussed above, β-defensins will need to be modified to have the appropriate therapeutic properties for use as an active pharmaceutical ingredient. This could include modifications to improve their metabolic stability, affinity, and specificity for particular targets.

In Vivo Efficacy: To prescribe β-defensins as preventive or therapeutic pharmaceutical agents, in vivo pharmacodynamic, pharmacokinetic, and toxicology studies need to be carried out. To our knowledge, no studies have evaluated the effect of β-defensins on mastitis in vivo in the presence of other biological components in the body, such as serum, milk, and microbiota. As the effectiveness and physiological activity of these peptides might differ from in vitro outcomes, further clinical trials are necessary to confirm their therapeutic effects.

Production Methods: The production and purification of β-defensins needs optimization. Peptides are categorized as small molecules and large biologics in some aspects, but they face challenges due to the intrinsic properties of amino acids, which can result in membrane impermeability and poor stability in vivo. Peptides can be synthesized, recombinantly expressed in bacteria, yeast, or mammalian cells, or extracted from natural sources. Each method has its own advantages and disadvantages in terms of cost, yield, quality, and scalability. To date, no study has compared the efficiency and feasibility of these methods for producing antimicrobial peptides on an industrial scale.

Delivery Vehicles: Peptides are typically not readily absorbed from mucosal surfaces, and they can be altered in the body by systemic proteases and enzymes, rapid metabolism, and opsonization. Therefore, appropriate delivery vehicles are essential to protect β-defensins from degradation and help them reach their target tissues. However, there are no studies currently available that determine the most effective delivery vehicle for these peptides concerning mammary gland infections.

Biological Reactions: The immunogenicity and allergenicity of β-defensins need to be assessed. As mentioned in previous studies, high concentrations of β-defensins adversely affect tissue epitheliocytes. They cause neutrophil migration, induce mucus hypersecretion, provoke mast cell degranulation, and increase vascular permeability [108]. Consequently, when evaluating the contribution of AMPs to allergic inflammation, it can be concluded that an excessive quantity of AMPs can exacerbate severe pathological changes in the organs.

## 9. Future directions

Mastitis is a complex disease that requires a comprehensive understanding of β-defensins in the bovine mammary gland. This review highlights knowledge gaps, proposing areas for future research. Filling these gaps can improve our understanding of the role of β-defensins in the bovine mammary gland. This will aid efforts to enhance immunity, treat mastitis, and explore alternative antimicrobial approaches. For future directions in this subject area, the following suggestions have been proposed:

1. Explore the effects of minerals and vitamins on the expression of β-defensins in the mammary gland.
2. Evaluate the potential immunomodulatory effects of β-defensins in the mammary gland.
3. Examine the response of β-defensins to other mastitis-causing pathogens ex vivo, including *Streptococcus uberis*, *Streptococcus dysgalactiae*, and *Klebsiella*.
4. Investigate the potential utilization of the MAPK pathway by other mastitis-causing pathogens, such as *E. coli*, to induce the expression of β-defensins.
5. Investigate the influence of hormones, for example, progesterone and calcitonin, on the expression of β-defensins.
6. Assess the possible effect of environmental factors, such as heat stress, on the expression of β-defensins.

## 10. Conclusions

In conclusion, this review provides a comprehensive overview of the current knowledge on β-defensins in the bovine mammary gland, highlighting their role as important AMPs involved in local defense against mastitis-causing pathogens. The increased expression of β-defensins in response to these pathogens suggests a potential innate immune mechanism for combating microorganisms in the mammary glands. While the TLR/NF-kB pathway has been implicated in β-defensin expression, it is crucial to recognize that additional pathways, such as MAPK and Oct-1, can contribute to their induction. Despite the therapeutic promise of β-defensins, there are still many knowledge gaps to address, such as their modification and strengthening and the selection of appropriate delivery vehicles, before their clinical application can be realized. Although several studies have investigated the effects of various factors, including plant-derived compounds and fatty acids on β-defensin expression in the mammary gland, many areas remain to be elucidated. By embracing the aforementioned suggestions and continuing to explore these research directions, we can advance the development of targeted strategies aimed at boosting the immune system, preventing and treating mastitis, and fostering the creation of alternative antimicrobials.

## Supplementary Materials

The following supporting information can be downloaded at: https://www.mdpi.com/article/10.3390/ani13213372/s1, Table S1: The impact of various pathogens or derived virulence factors on the expression of β-defensins’ genes and proteins, as well as the tests employed in different studies.
