# Supplementary material for: Expression, Regulation, and Function of β-Defensins in the Bovine Mammary Glands: Current Knowledge and Future Perspectives
Source: Animals (Basel). 2023 Oct 31;13(21):3372. doi: 10.3390/ani13213372 (PMC10650070; doi:10.3390/ani13213372)
Supplement: Supplementary file 1 [file animals-13-03372-s001.zip › animals-2652245-supplementary.pdf]

Table S1. The impact of various pathogens or derived virulence factors on the expression of β-defensins' genes and proteins, as well as the tests employed in different studies.

Study	Pathogen (or derived virulence factor)	Evaluated β-defensins[1]	Effect	Test	Sampling sites/ Evaluated cells	Study type
[19]	Subclinical mastitis	LAP<br>DEFB1<br>BNBD3	detected	RT-PCR[2]	Parenchymal and cistern tissue	*in vivo*-N[3]
	Purulent mastitis caused by *Actinomyces pyogenes*	LAP<br>DEFB401<br>BNBD9<br>BNBD12				
	Bloody mixed infection	DEFB1				
	Affected udder half, mixed infection	LAP<br>BNBD9				
	Purulent mixed infection	LAP				
[28]	*S. uberis*	LAP	↑[4]	In situ hybridization/ RT-PCR	Epithelium from the cisternal, alveolar, and peripheral regions	*in vivo*-E[5]
	Staphylococcus species<br>*Corynebacterium* species<br>*S. aureus*	LAP	↑	In situ hybridization/ RT-PCR	Epithelium from the cisternal, alveolar, and peripheral regions	*in vivo* -N
[26]	*A. pyogenes*<br>*E. coli*<br>*S. aureus*<br>*S. dysgalactiae*<br>*S. canis*<br>*S. uberis*	BNBD5	↑	In situ hybridization/ RT-PCR	Deeper area of udder quarter/mammary epithelial cell	*in vivo* -N
[52]	LPS<br>LTA	β-defensin (β -defensin sequences revealed the presence of LAP and TAP)	↑ (Response to LTA was lower)	qRT-PCR[6]	BMECs[7]	*ex vivo*
[100]	*S. aureus*	β-defensins	↑	RT-PCR	BMECs	*ex vivo*
[56]	*E. coli*	β-defensins (universal defensin)	↑	qRT-PCR	A deeper area of the udder quarter, 10 cm dorsal of the milk cistern/ regional udder lymph nodes and peripheral lymph nodes	*in vivo*-E
		BNBD5	↑			
	S. aureus	β-defensins (universal defensin)	↔	qRT-PCR		

		BNBD5	↔			
	E. coli	LAP	Induced	IHC	BMECs	*in vivo*-E
[61]	*E. coli*	LAP<br>DEFB1<br>BNBD4	↑	Affymetrix DNA-microarrays<br>qRT-PCR	BMECs	*ex vivo*
[51]	LPS	LAP	↑	ELISA	Milk	*in vivo*-E
[11]	Live *S. aureus*	TAP	↓	RT-PCR	BMECs	*ex vivo*
	Killed *S. aureus*		↑			
[89]	*S. aureus*	TAP<br>B-defensin	↔	RT-PCR	BMECs	*ex vivo*
[50]	*E. coli*	LAP<br>TAP<br>DEFB1	↑	Microarray analysis	Teat cistern lobulo-alveolar region	*in vivo*-E
[53]	LPS	LAP	↑	qRT-PCR	BMECs	*ex vivo*
[68]	*S. aureus*	TAP	↓	RT-PCR	BMECs	*ex vivo*
[48]	*S. aureus*	DEFB1<br>DEFB4<br>DEFB5	↑	qRT-PCR	Alveolar, ductal, gland cistern and teat canal regions	*in vivo*-E
		LAP<br>TAP	↔			
[90]	LPS	LAP	↑	RT-PCR	BMECs	*ex vivo*
[97]	*S. aureus*	TAP<br>BNBD5	↑	qRT-PCR	BMECs	*ex vivo*
[27]	*S. aureus*	LAP<br>TAP<br>DEFB1<br>BNBD5<br>BNBD10	↑	qRT-PCR	BMECs	*ex vivo*
[98]	*S. aureus*	LAP<br>TAP<br>DEFB1<br>BNBD4<br>BNBD5	↑	qRT-PCR	BMECs	*ex vivo*
		BNBD10	↔			

[74]	*E. coli*<br>*S. aureus*	BNBD5	↑ (Response to *S. aureus* was lower)	qRT-PCR	BMECs	*ex vivo*
[18]	*S. aureus*<br>*S. bovis*<br>*S. dysgalactiae*<br>*E. coli*	LAP	↑	ELISA	Milk	*in vivo* -N
[79]	*E. coli*<br>*S. aureus*	LAP<br>TAP	↑	Microfluidic high-throughput RT-qPCR	BMECs extracted from milk	*ex vivo*
[40]	*S. aureus- coagulase- positive and coagulase-negative*	LAP<br>DEFB1<br>BNBD4<br>BNBD5<br>BNBD10	↑	qRT-PCR	Parenchyma	*in vivo* -N
		TAP	No detected			
[75]	*S. aureus*	LAP<br>TAP<br>BNBD5	↑	qRT-PCR	BMECs	*ex vivo*
		BNBD10	↔			
[80]	LPS	LAP<br>TAP<br>EBD<br>BNBD1<br>BNBD5<br>BNBD3<br>BNBD4<br>BNBD6<br>BNBD7<br>BNBD10<br>BNBD13	↑	qRT-PCR	BMECs	*ex vivo*
[66]	*S. aureus*	LAP<br>TAP<br>DEFB1<br>BNBD4<br>BNBD5	↔	qRT-PCR	BMECs	*ex vivo*

		BNBD10				
[62]	*E. coli*	LAP	↑	qRT-PCR	The teat cistern	*in vivo*-E
	S. aureus		↔			
[64]	LPS	LAP	↑	qRT-PCR	Sections of heifer udders	*ex vivo*
	LTA<br>*S. aureus*		↔			
[84]	*S. aureus*	TAP<br>BNBD5	↑	qRT-PCR	BMECs	*ex vivo*
[86]	*S. aureus*	DEFB1<br>TAP<br>LAP	↑	qRT-PCR	BMECs	*ex vivo*
[81]	*E. coli*	BNBD5	↑	qRT-PCR	BMECs	*ex vivo*
[83]	LPS	BNBD5	↑	ELISA	BMECs	*ex vivo*
			↔	qRT-PCR	BMECs	*ex vivo*
	E. coli	TAP	↑	qRT-PCR		
		BNBD	↔			
[59]	Clinical mastitis	DEFB1<br>DEFB4A<br>DEFB7<br>DEFB10	↑	RNA sequencing	Circulating leukocytes	*in vivo* -N
	Subclinical mastitis	DEFB1<br>DEFB4A<br>DEFB7<br>DEFB10	↔			
[60]	*E. coli*	β-defensins	↑	RNA sequencing	Circulating Leukocytes	*in vivo* -N
[67]	*S. aureus*	LAP	↔	qRT-PCR	Precision-cut bovine udder slices/BMECs	*ex vivo*
[49]	Clinical mastitis<br>(*E. coli*<br>*S. uberis*)	DEFB4	↑	ELISA	Serum	*in vivo* -N
			↔		Milk	
	Subclinical mastitis<br>(*S. aureus*<br>*S. uberis*<br>*S. dysgalactiae*)		↓		Serum	
			↓		Milk	

[1] LAP: Lingual antimicrobial peptide; TAP: Tracheal antimicrobial peptide; BNBD: Bovine neutrophil β-defensins [Alternative names of BNBD1-13 genes are DEFB1-13.
[2] real-time PCR. [3] Incidence of infection naturally. [4] ↑: Upregulated; ↔: No change; ↓: Downregulated. [5] Incidence of infection experimentally. [6] quantitative real-time PCR.
[7] BMECs: Bovine mammary epithelial cells.
